# Lung Cancer Screening and Epigenetics in African Americans: The Role of the Socioecological Framework

**DOI:** 10.3389/fonc.2019.00087

**Published:** 2019-03-12

**Authors:** Karriem Sadot Watson, Alicia Hulbert, Vida Henderson, Ifeanyi Beverly Chukwudozie, Lisa Aponte-Soto, Lane Lerner, Erica Martinez, Sage Kim, Robert A. Winn

**Affiliations:** ^1^Cancer Center, University of Illinois at Chicago, Chicago, IL, United States; ^2^Department of Surgery, University of Illinois at Chicago, Chicago, IL, United States; ^3^Division of Health Policy and Administration, School of Public Health, University of Illinois at Chicago, Chicago, IL, United States; ^4^Division of Pulmonary, Critical Care, Sleep and Allergy, University of Illinois at Chicago, Chicago, IL, United States

**Keywords:** epigenetics, socio ecological model, disparities, African Americans, lung cancer

## Abstract

Lung cancer is the leading cause of cancer morbidity and mortality in the U.S. and racial/ethnic minorities carry the greatest burden of lung cancer disparities with African Americans (AAs) impacted disproportionately. Inequities in lung cancer health disparities are often associated with multiple bio-behavioral and socio-cultural factors among racial/ethnic minorities. Epigenetic research has advanced the understanding of the intersectionality between biological and socio-cultural factors in lung cancer disparities among AAs. However, gaps exist in the engagement of diverse populations in epigenetic lung cancer research, which poses a challenge in ensuring the generalizability and implementation of epigenetic research in populations that carry an unequal cancer burden. Grounding epigenetic lung cancer research within a socio-ecological framework may prove promising in implementing a multi-level approach to community engagement, screening, navigation, and research participation among AAs. The University of Illinois Cancer Center (UI Cancer Center) is employing an evidence–based (EB) model of community/patient engagement utilizing the socio-ecological model (SEM) to develop a culturally sensitive epigenetic lung cancer research program that addresses multiple factors that impact lung cancer outcomes in AAs. By implementing epigenetic research within a group of Federally Qualified Health Centers (FQHCs) guided by the SEM, the UI Cancer Center is proposing a new pathway in mitigating lung cancer disparities in underserved communities. At the *individual level*, the framework examines tobacco use among patients at FQHCs (*the organizational level*) and also tailors epigenetic research to explore innovative biomarkers in high risk populations. *Interpersonal interventions* use Patient Navigators to support navigation to EB tobacco cessation resources and lung cancer screening. *Community level* support within the SEM is developed by ongoing partnerships with local and national partners such as the American Lung Association (ALA) and the American Cancer Society (ACS). Lastly, at the *policy level*, the UI Cancer Center acknowledges the role of policy implications in lung cancer screening and advocates for policies and screening recommendations that examine the current guidelines from the United States Preventive Services Task Force (USPTF).

## Introduction

Lung cancer remains the leading cause of cancer mortality in the United States (U.S.) with a projected estimate of 234,030 new cases and 154,050 deaths from lung cancer in 2018 (1, 2). Despite recent declines in lung cancer mortality rates, inequities persist across racial and ethnic groups (2, 3). African Americans (AA) are disproportionately affected by lung cancer, and suffer greater morbidity and mortality than any other racial/ethnic group (4). Inequalities in lung cancer also exist among medically underserved communities (2, 3). The majority of medically underserved individuals in the U.S. receive their healthcare from Federally Qualified Health Centers (FQHCs), which are funded through the Health Resources and Services Administration (HRSA) under the Public Health Service Act (PHSA) Section 330 (5). FQHCs are charged with providing primary care including cancer screening and prevention services to populations that live in areas designated as medically underserved (6, 7). As one of the largest providers of safety net healthcare services in the U.S., FQHCs served over 25 million individuals in 2016 (5). While FQHCs often function as community clinics and serve a diverse and heterogeneous patient population, the majority of patients seen in FQHCs are from lower socioeconomic status (SES) and represent racial and ethnic minorities (5, 7).

FQHCs also provide care to a large number of smokers who meet criteria for lung cancer screening (7, 8). However, there are multiple challenges in implementing lung cancer screening within FQHCs that are both biological and socio-cultural (2, 6, 7). First, while there is a significantly high percentage of AAs who receive care at FQHCs, AAs show one of the lowest adherence rates to lung cancer screening (2). Second, while tobacco is a known risk factor for lung cancer, a disproportionate number of AA women die from lung cancer, despite having lower rates of smoking. This indicates that other biological, genetic, or environmental factors may contribute to lung cancer outcomes, which interact with gender and race/ethnicity (2, 9). Smoking, environmental, social, and economic neighborhood context are known to impact lung cancer disparities among AAs. Data from the National Health and Nutrition Examination Survey (NHANES) showed that although exposure to second hand smoke (SHS) was on the decline, AAs and groups living below the poverty level are still disproportionately affected by SHS (10). Similarly, racial/ethnic minority communities are more likely to be exposed to a built-environment that may result in increased lung cancer risk (11). Consequently, there is urgent need for conceptual/analytic models that examine multi-level factors for lung cancer incidence and mortality, including biological, environmental, and socio-cultural factors; and that particularly focus on identifying sources of disparities in lung cancer.

The study of epigenetics and social epigenomics enables researchers to understand the complex intersectionality of biology and socio-cultural factors such as diet, stress, built environment and cancer development and progression (9). However, conceptual models that examine associations between gene expression and multi-level social, environmental, and structural risk factors are needed to understand how health inequalities are produced and reproduced among those underserved, such as those seen and treated at FQHCs including AAs. In response to addressing risk factors that impact cancer outcomes in its catchment area, the University of Illinois Cancer Center (UI Cancer Center) has developed a multi-level approach to cancer screening, prevention and education embedded within a socio-ecological theoretical framework. The UI Cancer Center asserts that we can successfully respond to the intersection of biological and socio-cultural factors that contribute to cancer outcomes by addressing barriers and facilitators that affect cancer outcomes in underserved populations through a multi-level approach that aligns prevention, screening, navigation and epigenetic research.

## Screening in High Risk Populations

The National Lung Screening Trial (NLST) funded by the National Cancer Institute (NCI NCT00047385) demonstrated that lung cancer screening with the use of low-dose computed topography (LDCT) resulted in a reduction of lung cancer mortality (8). As a result, multiple professional organizations and policy leaders, including the USPSTF, advocate for LDCT screening for high-risk populations. While the results of the pivotal NLST trial demonstrated a 20% reduction in lung cancer mortality due to screening with LDCT, there is concern about the generalizability of the findings in low-resource and high-risk populations. It important to note that the sample in the NLST was predominately comprised of Non-Hispanic White (NHW) patients (7, 12). Although lung cancer results in an elevated mortality in AAs, only 4% of participants in the NLST were AAs (total 53, 542, 4% AAs, 5% other racial groups and 91% NHW) (8, 12). Additionally, the AA population included in the trial were more likely to be of younger age, had lower pack years, were more likely to be current smokers, and had lower SES and educational levels along with an increased likelihood of multiple co-morbidities (7).

**Figure 1:**
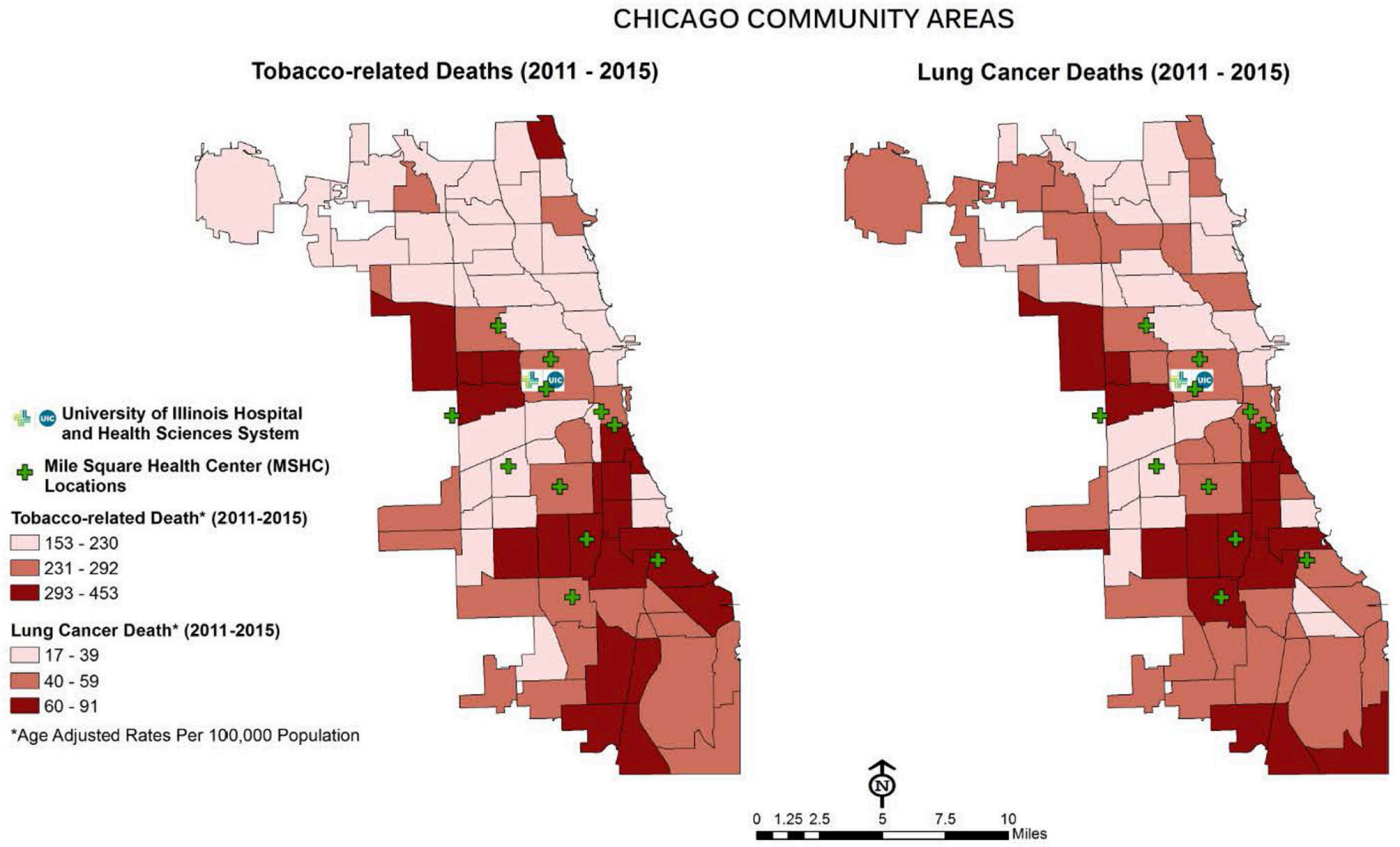
Tobacco-related and lung cancer death in Chicago.

The Mile Square Health Center (MSHC) is a network of 11 (FQHCs) providing comprehensive, high quality health services through the continuum of care (primary, preventative and specialty care, women's health, vision and dental care). MSHC is the third oldest FQHC in the U.S.; it opened in 1967 to address the needs of Chicago's public housing residents (13). Today, almost 40,000 patients, nearly three-quarters who are AA (74%), call MSHC their medical home. Co-owned and operated by UI Health, MSHC is one of the few FQHCs in the nation embedded within a health system, enabling seamless, comprehensive care coordination. MSHC clinics are located in neighborhoods carrying a disproportionate burden of tobacco use and elevated morbidity and mortality associated with chronic conditions, including cancer: Near West, Back of the Yards, Englewood, and South Shore (see Figure 1). Tobacco use in the MSHC catchment area exceeds the national average for all smokers across racial and ethnic groups with 40% AAs and 25% Latinx (14). MSHC reaches patients at educational, faith-based, and neighborhood events, by providing health resources, developing new jobs, and engaging the community in identifying and prioritizing their health needs.

MSHC's strong and positive presence in the communities it serves is evidenced by its cancer prevention initiatives, which reached more than 2,000 new patients in 2016. The “Mile Square Smoking Cessation Program” (MI-QUIT), funded by the March of Dimes Foundation, was launched in 2014. Originally, the objective of MI-QUIT was to focus on smoking cessation solely among women of reproductive age. However, the burden of chronic conditions experienced by tobacco users within the MSHC patient population extended beyond the original focus of the program. To respond to identified health disparities related to higher rates of tobacco use in the MSHC patient population, the funder approved an expansion of MI-QUIT to navigate all persons 18 and older to cessation services, with a dual emphasis on women of reproductive age and older adults at elevated risk of cancer, cardiovascular disease, diabetes, and other chronic conditions. Since the program's inception from 2014 to 2016, MI-QUIT navigated ~576 high risk patients to cessation services. In 2017, the MI-QUIT project was then funded by the Chicago Department of Public Health (CDPH) to address tobacco cessation in some of Chicago's most underserved communities. From 2017 to 2018, the MI-QUIT program navigated 327 tobacco users within the MSHC network to EB tobacco cessation services supported by training by the American Lung Association (ALA). Cessation services include lay and clinical patient navigation to care coordination, tobacco cessation motivational interviewing, facilitated EB tobacco cessation support group, and nicotine replacement therapies (NRTs).

## Multi-Level Approach to Tobacco Cessation and Lung Cancer Screening

**Figure 2:**
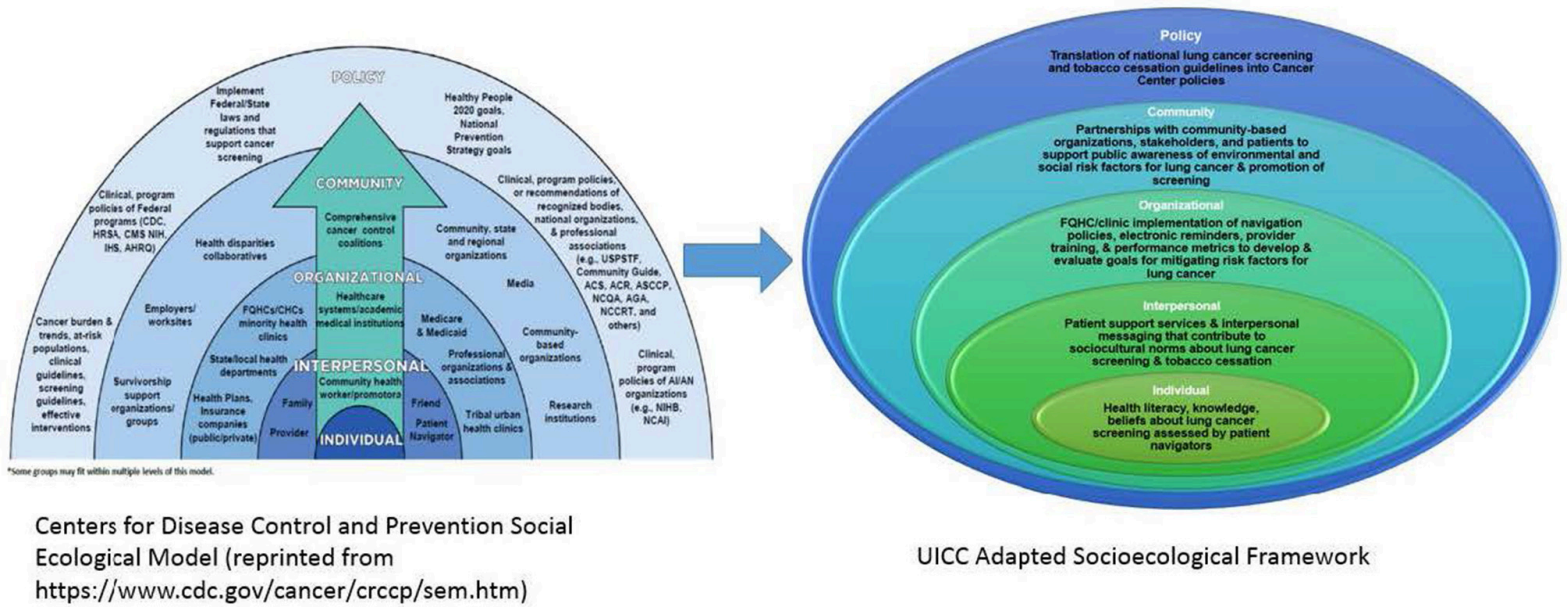
UICC social ecological framework for lung cancer screening and tobacco cessation adapted from CDC social ecological framework.

Disparities in lung cancer screening and tobacco cessation in racial and ethnic minorities, particularly in AA communities, is a multi-layered health inequity. Therefore, a multi-level solution is needed to examine the barriers and facilitators to lung cancer screening in high risk AA populations (15). The UI Cancer Center has adapted the Centers for Disease Control and Prevention (CDC) social ecological model (SEM) of health promotion for Colorectal Cancer Control Programs (CRCCP) to guide its community based lung cancer screening and tobacco cessation program with a focus on epigenetic research and patient navigation (see Figure 2). The multi-levels of the CDC's SEM provide an opportunity to examine lung cancer screening and tobacco cessation at the *individual, interpersonal, organizational, community*, and *policy* levels 16), resulting in increased access, detection, and tobacco cessation as evidenced by the MI-QUIT program.

### Individual Level

At the individual level, patient navigators play an integral role in accessing patients' knowledge and beliefs about lung cancer risk factors and screening (16). Patient navigators utilize a motivational interviewing model to assess patient's knowledge, health literacy levels, intentions, and understanding of risks and benefits related to lung cancer screening (17). Communication is tailored to each patient based on these assessments to ensure cultural and linguistic appropriateness, increasing patients' capacities to be active partners in shared decision making (SDM). As a result of the NLST, the USPSTF requires that SDM be documented for lung cancer screening to ensure that providers and patients have engaged in dialogue to support a patients' decisions to receive LDCT for lung cancer screening (18).

### Interpersonal Level

The second tier of the CDC's SEM model represents the interpersonal level. At this level, we aim to *facilitate* and minimize individual level barriers that patients may face through promotion of patient support services (7). This second level of the SEM highlights the role of *friends, family, health care providers, community health workers, and patient navigators* that can assist with developing and delivering interpersonal messages and services that promote and increase lung cancer screening rates, tobacco cessation rates, and address or mitigate barriers to screening that result from socio-cultural norms or logistical barriers (17). Cultural norms among AA communities known to impact lung cancer screening include fatalistic beliefs about lung cancer, medical mistrust, fears of racist conspiracy, and overall negative beliefs about cancer. These cultural norms may be mitigated at the Interpersonal level through proper messaging and support from patient navigators (19). MI-QUIT patient navigators assess barriers to screening that patients may have and work directly with patients in linking them to needed resources or services. Navigators also provide reminders to patients to improve screening compliance and address cultural factors that may impact screening behavior. Additionally, navigators provide cancer education, screening, and prevention materials. Navigators also work closely with providers to coordinate proper referrals for cessation services and lung cancer screening. The patient navigator-patient interactions coupled with education and culturally appropriate tailored services build trusting relationships that in turn increase a patient's likelihood to participate and complete screening, and follow up care (20). The partnership that navigators have with providers at each of the MSHC clinics also enhances the outcomes.

### Organizational Level

The third tier of the adapted model highlights the organizational level. This level examines what can be implemented at the systems level within FQHCs and other clinical settings that serve high risk, low-resource populations to support activities at the interpersonal level. For example, the utilization of electronic medical records (EMR) to develop client and provider reminders, implement provider level training, or utilize the Uniform Data Systems (UDS) metrics to develop and evaluate goals for mitigating risk factors for lung cancer such as tobacco cessation occur at this level (21, 22). Additional efforts at the *organizational level* may involve the development of a FQHC policy that highlights the need for and facilitates multi-level screening and navigation, integrating and evaluating EB practices through implementation science, conducting evidence based, clinic-level interventions, and fostering epigenetic research to address the multiple factors that impact lung cancer outcomes in high risk populations (23). The MI-QUIT program implements a number of activities to improve lung health outcomes at the organizational level. MI-QUIT patient navigators ensure that patients who are eligible for insurance coverage receive navigation to enrollment specialists and utilize the EMR system to properly navigate patients and monitor outcomes. Additionally, patient navigators inform providers of barriers patients encounter and collaborate with providers and administrative staff to address issues. For example, navigators partner with clinical staff to ensure that patients who need NRT support and other cessation support group services have access without the burden of costs. Providers and staff within the UI Health/MSHC system work closely and in partnership to streamline screening services and minimize potential healthcare system and clinic-level barriers to screening.

### Community Level

The community level is the fourth level of the adapted CDC SEM model. It demonstrates the dual role that FQHCs play by having the ability to function both at the organizational level and at the community level. The community level is especially important for the UI Cancer Center, which has a “bench to community model” of cancer care that moves beyond traditional engagement to support bi-directional engagement of both patients and community stakeholders. The UI Cancer Center and MSHC have built community coalitions and collaborations with national organizations such as the American Lung Association (ALA) and the American Cancer Society (ACS) Lakeshore Division in its cancer prevention and control efforts (24). The ALA has been a vital partner in supporting initiatives at multiple levels of the SEM, such as providing patient navigator and provider level training for tobacco cessation. Partnerships between local health departments (i.e., Chicago Department of Public Health-CDPH), FQHCs and academic entities (e.g., schools of public health) can further support community and public awareness of risk factor reduction for lung cancer and promote the awareness of screening and screening recommendations. Additionally, MI-QUIT promotes collaboration with partners at community wide educational campaigns including *Nobody Quits Like Chicagoland* and other lung cancer prevention and smoking cessation events. As part of our community level partnership, MI-QUIT coordinates dissemination of educational information to various community based organizations (CBOs), such as local tribal health clinics and LGBTQ organizations. Patient navigators also facilitate community cancer screening and prevention engagement through health fairs sponsored by local CBOs and elected officials. Partnerships with the CDPH provide resources to support EB navigation to the Illinois Tobacco Quitline and other local and statewide resources for tobacco cessation (25).

### Policy Level

The fifth level of the SEM is represented by the policy level. The *Policy Level* demonstrates a similar dual role that FQHCs play at both the interpersonal and community level, community stakeholders, CBOs and public health partners also play vital roles at both the community and policy levels. One of the aims of the Office of Community Engaged Research and Implementation Science (OCERIS) which sits within the Cancer, Prevention and Control (CPC) program of the UI Cancer Center is to identify and develop health policy and research priorities that are relevant to the catchment population, MSHC, community agencies, and other health delivery stakeholders. The SEM places a much needed focus on the ability to translate national lung cancer screening recommendations and evidence based guidelines for tobacco cessation into local cancer center policies supported by multiple stakeholders. Additionally, a critical examination of existing policies also informs an important research framework that assesses the applicability and generalizability of existing guidelines and recommendations for high risk populations. For example, the existing USPSTF lung cancer screening guidelines were based on the results of the NLST, which were biased by the underrepresentation of racial and ethnic minorities. Additionally, the *policy level* of the SEM also addresses environmental factors. Underserved communities and predominantly racial/ethnic minority communities, including AA communities, are known to have greater environmental exposure to air pollution and other environmental carcinogens (10). From a socio-cultural perspective, the *policy level* also examines socio-environmental factors such as crime, community support and collective efficacy and built-environmental factors such as food deserts, lack of walkable space, and other physical attributes of the environmental spaces and their role as barriers to lung cancer screening in AA communities (11). Deploying a SEM allows the UI Cancer Center to develop research and screening projects that reflect the behaviors and needs of its cancer center catchment population which differs from the research participant demographic of the NLST (8, 26). Early outcomes of the MI QUIT Program at the *policy level* include recent collective advocacy between the UI Cancer Center and ACS to provide educational and EB data to elected officials in Illinois to support increasing the minimum age for tobacco use, *Tobacco 21*.

Analysis of groundbreaking studies like the NLST show that while advances have been made in reducing lung cancer mortality, the far reaching implications of these advances to racial and ethnic minorities, particularly AA communities and other underserved populations, may be limited (2). In addition to the lack of diversity in research participation, the NLST was also limited in its assessment of social determinants of health and other socio-cultural factors that may directly interact with biological factors that impact lung cancer outcomes. Exploring epigenetic research utilizing a socio-ecological framework may have promise in addressing the many conundrums that exist among cancer disparities in underserved populations (9). Across the SEM, epigenetic research may provide key information into the interplay of individual behaviors such as tobacco use and exposure and community level exposures such as environmental toxins and exposures to stress across *individua*l and *inter-personal levels* (23).

## Epigenetics in Lung Cancer Disparities

The term “epigenetic” refers to the change in gene expression that is mediated by acquired and heritable mechanisms without alterations in the primary nucleotide genetic sequence. Acquired epigenetic changes can promote initiation and progression of cancer by modulating gene expression (27–32). There are three main, inter-related types of epigenetic inheritance: DNA methylation, genomic imprinting and histone modification. The most studied epigenetic mechanism is the methylation of genomic promoter regions. Methylation in cancer is an example of epigenetic dysregulation, with both hypomethylation and hypermethylation having significant roles in cancer molecular development and progression (33–38).

Although disproportionate lung cancer mortality rates among AA persist, few studies have investigated specific changes in gene methylation related to lung cancer in racial/ethnic groups. Most biomarker-specific studies have either ignored racial/ethnic specific differences entirely or feature dense NHW sample cohorts. While it is likely that most pathways may remain consistent between groups, it is possible that exposure and access to different factors, including different biological, environmental, and socioeconomic conditions, may contribute to differential epigenetic changes, leading to racial disparities in lung cancer outcomes among AAs (39–41). And yet, specific mechanisms through which how neighborhood conditions may contribute to epigenomic changes and gene expression have been far less explored (42).

Despite having greater lung cancer exposure and unexplained associations between biological and socio-environmental factors, AAs are insufficiently represented in current translational and epigenetic research (43–45). Recent research in comparative epigenetics in the U.S. reveals a spectrum of promoter methylation across racial/ethnic groups, the body of studies published in the literature about epigenetics in cancer in minority populations is scarce. Many studies often do not indicate race-specific changes in DNA methylation (42, 46, 47). However, there has been progress. Kwabi-Addo and co-workers examined the methylation pattern of six different genes (GSTP1, AR, RARβ2, SPARC, TIMP3, and NKX2-5) in prostate tissue specimens from AA and NHW males. They observed significantly higher methylation for all genes, except GSTP1, in the AA samples in comparison to that from NHW prostate cancer patients. In addition, two genes (NKX2-5 and TIMP3) were hyper-methylated in normal prostate tissue samples of AA racial background as compared to those from NHW (48). Wang et al. analyzed DNA methylation patterns in AA and NHW breast cancer patients (49). They found significant methylation differences in the promoter CpG island of the tumor suppressor gene, CDH13. AA patients' demonstrated increased hypermethylation compared to matched NHWs. This hypermethylation was found to be significantly associated with decreased breast cancer survival (49). Figueiredo et al. (50) found that global methylation was assessed via bisulfite pyrosequencing of long interspersed nuclear element-1 (LINE-1) from colon cancer biopsy samples. A trend of global hypomethylation was associated with race with AAs more hypomethylated than the NHW counterparts. In an epidemiologic study by Terry et al. differences in DNA methylation by race were observed, with AAs more likely to have lower levels of DNA methylation than NHWs or Hispanics (51). Sun et al studied smoking-related DNA methylation in AAs and found a trend of lower hypomethylation in AA women in factor II receptor-like 3 (F2RL3) and G-protein-coupled receptor 15 (GPR15) (52). Similarly, Dogan et al. analyzed DNA methylation in peripheral blood mononuclear cells from AA women and found significant loci in respect to smoking status, specifically, two aryl hydrocarbon receptor repressor (AHRR) gene loci (cg0557921 and cg23576855) and one GPR15 gene loci (cg19859270) (53). In agreement with Sun et al. these genes were found to be hypomethylated in AAs (53). Similarly, Philibert et al. found DNA demethylation at two different AHRR sites among AA male smokers (39). In a subsequent study, the investigators found increased rates of DNA methylation at AHRR were more pronounced among those who quit smoking in comparison to individuals who were unable to quit (40). These data provide evidence that methylation is race-specific with AAs exhibiting greater trends for smoking specific hypomethylation sites.

In relation to health disparities, the impact of social stressors (stress, starvation, domestic violence, veterans, genocide, war) has been shown to cause altered methylation of stress pathways (54). In breast carcinoma, hypomethylation of the glucocorticoid receptor (GR) gene was observed in breast carcinoma (55). In small-cell lung cancer, hypermethylation of the NRC31 promoter region of the GR gene was observed in a panel of 14 human SCLC cell lines (56). These findings all suggest that additional research needs to explore the role of epigenetics in lung cancer and other cancer disparities, particularly between and within racial and ethnic groups, with a focus on AAs.

Approximately a third of the U.S. population is comprised of racial/ethnic minorities, but recent reports suggest that minorities make up <18% of the patient population in clinical trials supported by the National Cancer Institute (NCI) and 17% of all Food and Drug Administration (FDA) clinical trial participants (57). Minority engagement in clinical trials is particularly low in cancer research (58, 59). This lack of diversity in research can lead to several problems including, but not limited to, questions about the generalizability of research findings, the accuracy of subgroup studies, misalignment of interventions, and unequal access to healthcare innovation (45, 60–64). The National Institute of Health (NIH), the FDA, and Medicare/Medicaid programs have sought to address underrepresentation of racial and ethnic minorities in research through novel funding initiatives in recent years that identify strategies for culturally appropriate recruitment and retention (45, 65–67), including the incorporation of patient navigation models (68) and education about research (69, 70). However, barriers to participation remain an important concern, both within the research environment and at the community level. These barriers may be mitigated by adopting the SEM in research design and implementation processes.

### Using the SEM to Address Lack of Engagement of Diverse Populations in Epigenetic Research

#### Individual Level

At the individual level, barriers for minority participation faced by *researchers* range from broad to specific challenges. Historical use and misuse of minority populations in research (e.g., the Tuskegee Syphilis study) have contributed to generational medical and research mistrust (71), lack of knowledge of research and medical jargon, language, literacy, health status, and not being included in the consent process (72). Barriers for *minority participants* themselves primarily center on historical misinformation and logistical issues that have contributed mistrust of biomedical research among minority, including AA communities at multiple levels. Namely, the U.S. Public Health Service Syphilis Study at Tuskegee and the stories of individuals such as Henrietta Lacks have impaired the relationship between the AA community and research groups (73).The perception that medical research is solely geared toward the benefit of NHWs or research institutions and exploits the AA community persists to this day (45, 74). Asian Americans, Hispanics, and Pacific Islanders exhibit similar mistrust which derives from cultural histories of exploitative studies, such as the forced sterilization of Native American and Puerto Rican women, or from their vulnerability in terms of immigration status (45, 75–78).

Additionally, cultural views and stigmas may prevent individuals from consenting to research studies. Recent reports have shown that the AA community is particularly concerned with investigations regarding genetics and mental illnesses (45, 79); and the Hispanic community is wary of HIV research (45, 80). Both of these trends are related to cultural perceptions of the related health conditions and each strongly impacts lung cancer related translational epigenetic studies.

Logistically, factors that impact low socioeconomic status groups create hurdles for underrepresented racial/ethnic minorities. Scheduling conflicts, issues related to child care, lack of transportation, lack of community or financial support structures all play a role in reducing minority participation in research at the *individual level*. Additional burdens such as interpreting lengthy or jargon-filled research documents reduce participation further (44, 45, 62, 64, 81). Engagement at each level of the SEM may prove effective in addressing historical mistrust and ongoing lack of diversity in clinical trials.

### Interpersonal Level

Similarly, at the interpersonal level, investigators encounter barriers in recruiting, enrolling, and retaining, racial and ethnic minorities in clinical and epigenetic trials. Provider and/or investigator biases including assumptions about the lack of interest or participation of racial and ethnic minority patients can contribute to lower enrollment rates (72). Lack of knowledge about how to approach different cultures can hold researchers back from effective or meaningful dialogues with groups. This gap in understanding can lead to ineffective communication, which hampers recruitment, enrollment, and retention (45, 62, 82, 83). Additionally, countering mistrust by establishing community trust and developing culturally informed strategies including meeting the participants' language and linguistic needs, which are fundamental to successful enrollment and continued adherence to study protocols, both require time and effort to establish (45, 64, 84–87). Ensuring that researchers, patient navigators, and providers mirror the patient population may also facilitate building trusting relationships with patients and improve unbiased recruitment because of the increased cultural sensitivity and understanding of cultural values (72). FQHCs can incorporate patient navigation models and collaborate with providers to increase patient knowledge and recruitment in research studies (68, 72).

#### Organizational Level

At the organizational level, FQHCs can develop inclusive policies in research and institute a community engaged research framework that can support participation of groups traditionally under-represented in research. Establishing a community board, a research council, and/or patient advisory group that engages patients as partners in the development, review, and approval of research projects that are implemented at FQHCs as well as in the decision making process for culturally tailored recruitment practices may be beneficial. These are strategies currently employed by the UI Cancer Center in partnership with the MSHC clinics.

### Community Level

At the community level, activities at the community level, such as the engagement of diverse stakeholders in community based dialogue about the importance of participation in epigenetic and lung cancer research can advance trust through the SEM. Language barriers also require the use of bilingual staff to access specific populations; however, the limited nature of grant funding forces hard decisions that can limit study scope. Similar issues such as the resources required to travel to and engage with specific populations can lead to restrictions in study scope (45, 64, 82, 83, 88–90).

The ultimate indicator of the impact of lung cancer screening and increasing engagement of diverse populations in epigenetic research is impacted by *policy level* changes. The UI Cancer Center collaborated with stakeholders from the Society of Behavioral Medicine (SBM) to develop a policy brief to highlight the importance of lung cancer screening using LDCT in high risk populations (18). Policy recommendations for providers and researchers encourage more funding toward development of research that examine lung cancer disparities in high risk populations (18). Additionally, policy recommendations that foster translational research such as epigenetic research may prove beneficial in addressing the biological and socio-cultural factors at play in lung cancer disparities.

### Advancing Diversity in Lung Cancer Research Through Community Based Epigenetic Research

In order to bring a diverse patient cohort including more AAs into translational epigenetic studies and pave the way for epigenetic screening in underrepresented communities, the UI Cancer Center has partnered with the Cancer Epigenetic Liquid Biology Program (CELLI) team to develop: (1) a diagnostic screening investigation that monitors non-cancer patients following their initial laboratory results and determines the strength of the correlation between positive epigenetic screening results and clinical diagnoses of cancer; and (2) a diagnostic validation investigation, which will compare laboratory results from cancer and non-cancer patients to determine if the strength of the positive predictive values and negative predictive values observed in a prior study with a non-diverse population hold true in a new more diverse patient population. Using a panel of DNA methylation based biomarkers, whose efficacy was previously established in a another study, the CELLI Team will collect blood and urine specimens from patients at three FQHCs in high risk communities, the Englewood, Back of the Yards, and Near West (Main) MSHC sites (46). Cell free DNA (cfDNA) from these circulating fluids will be isolated and undergo bisulfite conversion in preparation for epigenetic screening. This is a direct example of how epigenetic research can be advanced by building upon the socio-ecological framework, and advancing innovative biological breakthroughs.

## Conclusion

To deploy theory into practice, the UI Cancer Center is implementing a multi-level approach to address lung cancer screening within high risk AA and FQHC populations. Guided by the CDC's SEM, the UI Cancer Center utilizes Implementation and Dissemination Science to continue to implement both standard-of-care lung cancer screening and navigation along with innovative epigenetic and health disparities research (91). Early success to date, include engagement of more than 500 patients from the MSHC FQHC who were navigated to EB tobacco cessation and lung cancer screening using the revised SEM. This indicates the utility of the multi-level approach to address lung cancer disparities. Additionally, the implementation of the CELLI epigenetic lung cancer screening study within the MSHC FQHC also demonstrates the feasibility of tailoring epigenetic research to meet the needs of underserved racial and ethnic minorities, including AA populations. While previous large scale studies like the NLST made major advances in lung cancer research, several factors such as lack of participant diversity and limited focus on socio-cultural factors of the NLST may impact current gaps that exist in the lung cancer conundrum among racial and ethnic minorities, with a specific emphasis on AAs. Future lung cancer screening efforts of the UI Cancer Center will also deploy extensive questionnaires to assess individual and community level factors such as smoking behavior and access to EB tobacco cessation services and lung cancer screening. Future directions will also uncover the barriers among AAs to participating in epigenetics research. The use of the SEM will allow investigators at the UI Cancer Center to assess the multiple layers and levels that impact lung cancer disparities among its catchment. Epigenetic research rooted in an ecological model may serve as the bridge connecting the various factors that impact lung cancer outcomes in AA and other racial/ethnic minority communities.

## Author Contributions

All authors listed have made a substantial, direct and intellectual contribution to the work, and approved it for publication.

### Conflict of Interest Statement

The authors declare that the research was conducted in the absence of any commercial or financial relationships that could be construed as a potential conflict of interest.
